# Heuristic thinking and altruism toward machines in people impacted by COVID-19

**DOI:** 10.1016/j.isci.2021.102228

**Published:** 2021-02-23

**Authors:** Celso M. de Melo, Jonathan Gratch, Frank Krueger

**Affiliations:** 1 CCDC U.S. Army Research Laboratory, Playa Vista, CA 90094, USA; 2 USC Institute for Creative Technologies, Playa Vista, CA 90094, USA; 3 George Mason University, Fairfax, VA 22030, USA

**Keywords:** Computer Science, Human-Computer Interaction, Sociology

## Abstract

Autonomous machines are poised to become pervasive, but most treat machines differently: we are willing to violate social norms and less likely to display altruism toward machines. Here, we report an unexpected effect that those impacted by COVID-19—as measured by a post-traumatic stress disorder scale—show a sharp reduction in this difference. Participants engaged in the dictator game with humans and machines and, consistent with prior research on disasters, those impacted by COVID-19 displayed more altruism to other humans. Unexpectedly, participants impacted by COVID-19 displayed equal altruism toward human and machine partners. A mediation analysis suggests that altruism toward machines was explained by an increase in heuristic thinking—reinforcing prior theory that heuristic thinking encourages people to treat machines like people—and faith in technology—perhaps reflecting long-term consequences on how we act with machines. These findings give insight, but also raise concerns, for the design of technology.

## Highlights

• Participants engaged in a dictator experiment with humans and computers
• We measured impact of COVID-19 using a PTSD scale
• COVID-19 led to increased heuristic thinking, faith in, and altruism with computers
• These findings raise opportunities and concerns for the design of future technology

## Graphical abstract

**Figure:**
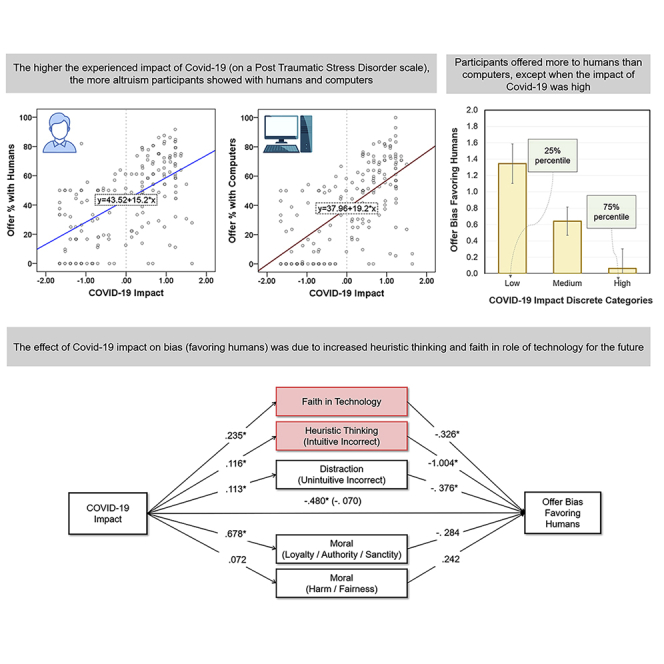

## Introduction

With the advent of autonomous technology (de Melo et al., 2019; Stone and Lavine, 2014; Waldrop, 2015)—e.g., automated vehicles, drones, robots, personal assistants, etc.—it is important we understand how to promote collaboration between humans and machines. Given that people lack knowledge and experience about how autonomous machines function, trusting and adopting machines can be challenging (Gillis, 2017; Lee and See, 2004). On the one hand, early work on human-computer interaction suggests that humans are prone to treat machines in a social manner, as a cognitive heuristic, just like they would other humans (Blascovich et al., 2002; Nass et al., 1994; 1999; Reeves and Nass, 1996; von der Pütten et al., 2010), and that these effects can be leveraged to create more effective applications. On the other hand, more recent work emphasizes that these tendencies are not as strong and there are important differences in the way people behave with humans when compared to machines (Gallagher et al., 2002; McCabe et al., 2001; Rilling et al., 2002; Sanfey et al., 2003). For example, in exact same decision tasks, people are less likely to follow social norms such as fairness with machines (de Melo et al., 2016; de Melo and Terada, 2019; Terada and Takeuchi, 2017). This difference can be problematic for the successful adoption of autonomous technology, as it imposes a limit on the amount of collaboration that can be achieved, especially when compared to human-human interaction. It is, thus, necessary to understand why these differences occur and how (or if) to overcome them. However, in the course of studying human behavior with machines, we recently noticed an unexpected source of individual variation: people impacted by COVID-19 were acting more altruistically with machines (see the supplemental information for more details on one of these exploratory studies). Here, we focus on this effect, seek to understand the mechanism underlying it, and articulate broader implications for our understanding of collaboration between humans and machines.

The COVID-19 pandemic has had profound health, economic, and social impact across the globe. At the time of the writing, there were over 101 million confirmed infection cases and over 2.19 million deaths worldwide. In the United States (US) alone, there were over 14 million confirmed infections and over 433,000 deaths (https://coronavirus.jhu.edu/map.html, accessed Jan-29, 2021). The pandemic has also caused a significant economic disruption, including due to lockdown measures imposed to contain the spread of the infection. One consequence of the social distancing measures imposed to contain the spread of COVID-19 was the (forced) adoption of technology to support remote social and professional activities. Technology, moreover, is also expected to continue playing an important role as economies re-open (e.g., to support contact tracing). Increased exposure and reliance on technology during the pandemic, therefore, may be contributing to changing people's attitudes toward technology and machines.

Changing behavior with machines is especially relevant at a time of unprecedented progress in artificial intelligence technology, including the emergence of autonomous machines that act on behalf of others. Human-machine interaction studies (in what has been called the Computers as Social Actors theory) show that, in social settings, people tended to engage with machines in a social manner (Nass et al., 1994; Reeves and Nass, 1996), for instance, showing politeness toward machines (Nass et al., 1999) or responding to their social cues (de Melo et al., 2014). The argument is that people resort to heuristic thinking and intuitively carry their experience from human-human interaction to human-machine interaction (Blascovich et al., 2002; Reeves and Nass, 1996) and that designers can use this theory to create more effective systems (e.g., Pak et al., 2012). Some researchers, moreover, argue that heuristic thinking can increase cooperation with others, as intuitive responses may have been shaped and internalized as social heuristics, by prior experience of cooperative interactions (Rand, 2016; Rand et al., 2014). Others, however, have questioned such a direct relationship between intuitive responses and increased cooperation—e.g., Verkoeijen and Bouwmeester (2014). Heuristic thinking, therefore, can play an adaptive role in helping humans make sense of machines. The more this type of thinking is encouraged, consequently, the higher social influences are machines expected to have on humans and the higher collaboration they are likely to motivate from humans.

However, despite treating machines as social actors, recent research suggests that humans still make important distinctions when engaging with machines, when compared to humans. This work shows that people can reach different decisions and show different patterns of brain activation with machines. For instance, Gallagher et al. (2002) showed that when people played the rock-paper-scissors game with a human, the medial prefrontal cortex is activated, a brain region that is consistently implicated in mentalizing (i.e., inferring of other's beliefs, desires, and intentions); however, no such activation occurred when people engaged with a machine that followed a known predefined algorithm. McCabe et al. (2001) found a similar pattern when people played the trust game with humans vs. machines, and others replicated this finding using prisoner's dilemma games (Kircher et al., 2009; Krach et al., 2008; Rilling et al., 2002). In economic exchange games, moreover, participants tended to show less cooperation, fairness, and altruism with machines when compared to humans (de Melo et al., 2016; de Melo and Terada, 2019; Terada and Takeuchi, 2017). These differences are problematic as they introduce an important barrier to collaboration with machines.

The COVID-19 pandemic may be inadvertently helping break these barriers to collaboration with machines. The pandemic is having a considerable impact on people's mental health, including post-traumatic stress disorder (PTSD), due to financial distress, social distancing, and uncertainty about the future (Pfefferbaum and North, 2020). Through the course of the pandemic, increased stress may lead to increased cognitive burden and, consequently, more heuristic thinking, including when engaging with machines. If heuristic thinking is truly at the heart of people's prosocial behavior toward machines, then increased heuristic thinking, in turn, may accentuate people's tendency to treat machines like humans and, consequently, encourage more favorable decisions with machines. To study this, we focus on “altruism” as a simple measure of social consideration for others (Forsythe et al., 1994)—however, see the supplemental information for a pilot study that looked at the impact of Covid-19 on reciprocity. When one behaves altruistically, one helps another at a cost to the self without getting a direct benefit from the interaction (e.g., donating money to a stranger). To measure altruism, we considered the dictator game (Forsythe et al., 1994), which is an economic decision-making task involving two players: a sender and a receiver. The sender receives an initial endowment—in our case, 12 tickets to a lottery worth $30—and then decides how many to give away, whereas the receiver has no say and must accept whatever was sent. Rational theory argues that there is no incentive to send anything and, thus, senders are expected to send zero tickets. Nevertheless, in practice, people offer an average of 10–25 percent of the initial endowment and, often, an offer of 50 percent is made (Forsythe et al., 1994; Henrich et al., 2001). Decisions in this game, thus, have been argued to reflect altruism as it rules out other motives for giving including, for example, the expectation of future reciprocity (Bolton and Ockenfels, 2000; Camerer, 2003). The dictator game, therefore, is ideal to study social behavior with machines, as the decision maker holds all the power. The main hypothesis in the paper is, thus, that people impacted by COVID-19 will be more altruistic toward machines.

This hypothesis is further supported by research suggesting that external events—e.g., natural disasters—can lead to increased reciprocity, trust, and altruism toward others. Research shows that natural disasters can improve social cohesion, trust, and altruism in affected communities, due to a need to cooperate to tackle the challenge and recover quickly (Calo-Blanco et al., 2020; Cassar et al., 2017; Toya and Skidmore, 2014; Whitt and Wilson, 2007). For instance, Chileans affected by the 2010 Maule earthquake were more likely to give to charity, engage in volunteering, and less likely to commit crimes (Calo-Blanco et al., 2020). In contrast, scarcity and competition for valuable resources can lead to reduced trust in some cases (Carlin et al., 2014; Hsiang et al., 2013). The specific socio-cultural context and prevalent institutions, thus, are important to understand the effect external environmental events have on human behavior. If the COVID-19 pandemic is causing people to show increased consideration for others, then it may lead them to make more favorable decisions to others. Moral theory further argues that increased empathy can lead to individuals to consider more distant others (Singer, 1981; Zaki, 2014). Beyond caring for the self and close family, individuals may be motivated to consider extended family, friends, communities, nations, and even non-human others (Graham et al., 2017; Waytz et al., 2019).

The COVID-19 pandemic is also changing our attitudes toward technology, which may lead to long-term effects in the way people engage with machines. Social distancing has forced individuals and businesses to adapt and experience life remotely and one consequence appears to be a greater appreciation for the role of technology to the future. In the stock market, for instance, whereas most other sectors were slower to recover, the tech sector remained mostly strong, suggesting that investors foresee a future where technology will play an increasingly important role (Wigglesworth, 2020). Growing consideration for the value of technology may induce a long-term motivation, perhaps even post-pandemic, to make more favorable decisions with machines.

The potential effect of COVID-19 on behavior with machines is, thus, motivated by three possible mechanisms. First, increased heuristic thinking may lead people to treat machines more like other humans. Second, increased empathy toward others may lead to increased moral consideration for non-human others, including machines. Third, increased faith in technology may lead to more altruistic decisions with machines. Here, we present an experiment that tests our hypothesis and teases apart these possible mechanisms.

**Figure 1:**
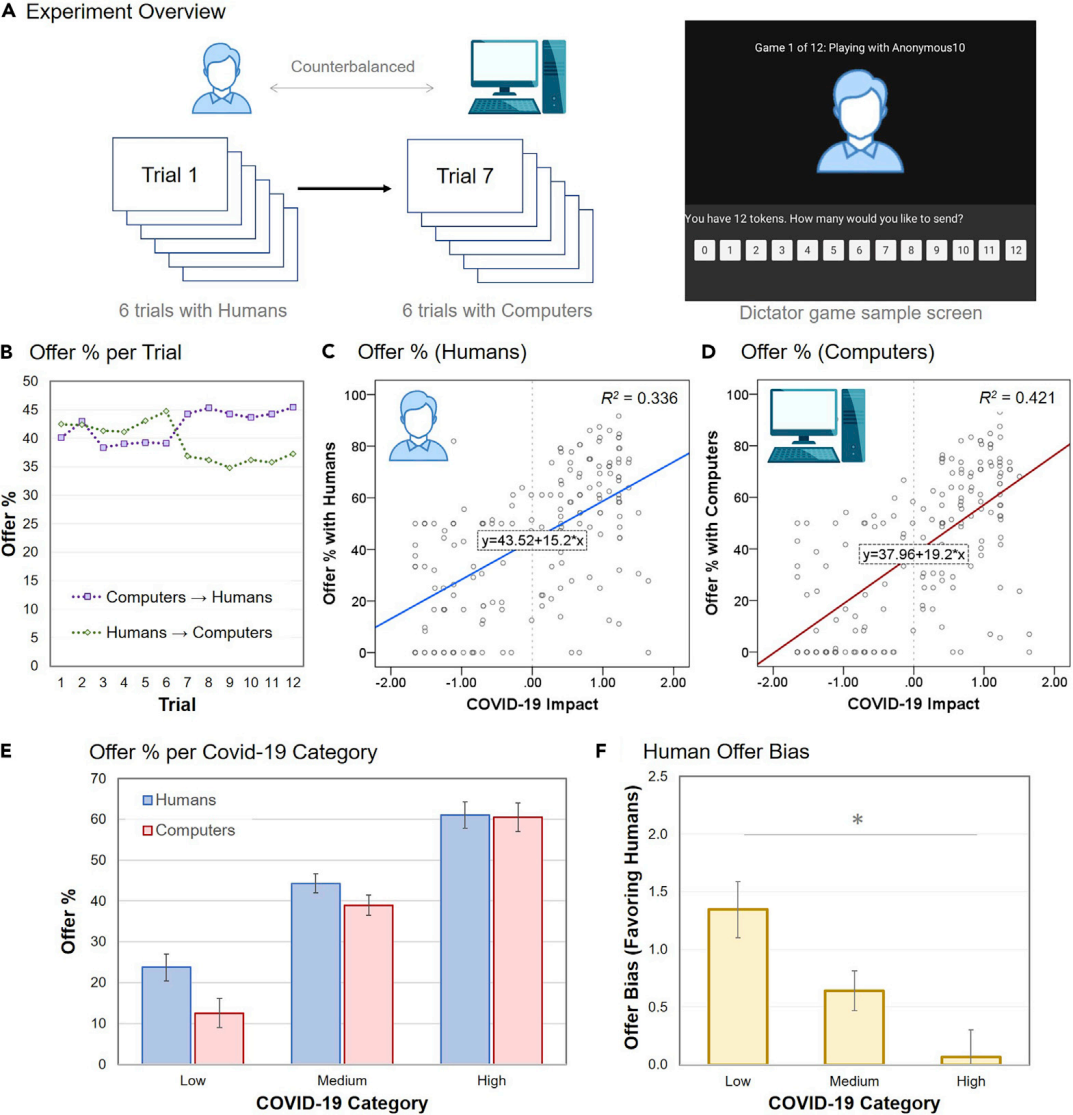
Experimental design and results for the dictator game (A) Experimental design overview. On the right, a screenshot of software is shown for the dictator game. (B) Offers (in percentage of initial endowment) for each trial, split by counterpart order. (C) Impact of COVID-19 increased offers (in percentage) with human counterparts. (D) Impact of COVID-19 offers (in percentage) with computer counterparts. Notice that the gradient for the linear fit was higher with the computer than with human counterparts. (E) Offers (in percentage) per COVID-19 category. Error bars show standard errors. (F) Participants impacted by COVID-19 showed a lower bias (i.e., offer to humans minus offer to computers) in favor of human counterparts than other participants. Error bars show standard errors. ∗p < 0.05.

Participants engaged in multiple trials of the dictator game as senders and were instructed that receivers would either be other participants or computers. Each trial was ostensible with a different (human or computer) counterpart, and the trials with each kind of counterpart were blocked (six trials with computers and six trials with humans), with the block order being counterbalanced across participants (Figure 1A). In reality, to maximize experimental control, participants always engaged with computer scripts. Participants were debriefed at the end of all procedures, and the experiments were approved by the University of Southern California's institutional review board. To minimize reputation effects, the experiment was anonymous, both with respect to other participants and experimenters. Please see the supplemental information for details on how this was accomplished and Video S1 for details on the experimental software.

Video S1. Software used in the dictator game experiment, related to Figure 1

A sample of 186 participants was recruited as senders for this experiment on Amazon Mechanical Turk. Prior research indicates that this online platform can yield high-quality data and successfully replicate behavioral results in traditional pools (Paolacci et al., 2010). Moreover, this pool allowed us to recruit a diverse sample from over 40 states in the US (see the supplemental information Transparent methods section and Figure S1 for details on participant samples' demographics).

To measure the impact of COVID-19, we adapted the abbreviated PTSD Checklist-Civilian 6-item scale for measuring PTSD in general medical settings (Lang and Stein, 2005). The instructions asked participants to rate on a 5-point Likert scale how much they experienced certain problems in the last month resulting from the pandemic (e.g., “Feeling very upset when something reminded you of the situation,” “Feeling distant or cut off from other people”). An individual is screened positive for PTSD if the sum of these items is 14 or higher—according to this procedure, 65.8% of the sample screened positive (see the supplemental information for sample distribution details for this scale and an analysis indicating a lack of influence of participants' political ideology on COVID-19 scores). For our formal analysis, we ran a principal component analysis with varimax rotation to reduce the scale to a single factor (Cronbach α = 0.939; see Table S1 in the supplemental information for factor loadings).

To get insight on mechanism, we asked participants to answer three subjective scales. Nass and colleagues claim that people treat machines in a social manner because they heuristically apply human social script (Nass and Moon, 2000; Nass et al., 1994; Reeves and Nass, 1996), although they never attempted to manipulate or measure a person's tendency to engage in heuristic thinking. To improve upon this, we adopted the cognitive reflection test (Frederick, 2005) to measure if those impacted by COVID-19 were engaging in reduced reflection, i.e., more intuitive thinking. This test consists of questions (e.g., “A bat and a ball cost $1.10 in total. The bat costs $1.00 more than the ball. How much does the ball cost?”) with an intuitive incorrect answer (10 cents) and a correct answer that requires increased reflection (5 cents). This scale provides a proxy for heuristic thinking by counting the intuitive incorrect answers (Toplak et al., 2011) (see Table 1 for details on this scale). Second, as argued above, it may be that those impacted by COVID-19 develop a growing appreciation for technology. So, we asked participants to rate five statements about their faith in technology, such as “Computer technology will change life for the better.” and “Computer technology advances will solve America's social and economic problems within the next ten years.” (see Table S2 in the supplemental information for full details on this scale). Finally, research suggests that those with higher moral foundations—especially in the care/harm and fairness foundations—will show higher consideration for non-humans; thus, we asked participants to answer the Moral Foundations Questionnaire (Graham et al., 2013) (see Table S3 for more details on this scale).

**Table 1:** The cognitive reflection scale

Question	Correct answer	Intuitive incorrect answer	(Unintuitive) incorrect answer
1. A bat and a ball cost $1.10 in total. The bat costs $1.00 more than the ball. How much does the ball cost (in cents)?	5	10	Anything else
2. If it takes 5 machines 5 min to make 5 widgets, how long would it take 100 machines to make 100 widgets (in minutes)?	5	100	Anything else
3. In a lake, there is a patch of lily pads. Every day, the patch doubles in size. If it takes 48 days for the patch to cover the entire lake, how long would it take for the patch to cover half the lake (in days)?	47	24	Anything else

## Results

We first looked at dictator game offers across trials for each of the counterpart order, as shown in Figure 1B. We ran an order × trial repeated measures analysis of variance (ANOVA), which revealed no effect of order (*F*(1, 184) = 0.507, p = 0.447), no effect of trial (*F*(11, 2024) = 0.851, p = 0.589), but a statistically significant order × trial interaction (*F*(11, 2024) = 8.669, p < 0.001, partial *η*^*2*^ = 0.045). This interaction reflects the switch at the seventh round, when participants started engaging with the other counterpart type, thus, supporting the effectiveness of the experimental manipulation. We then looked at the effect of COVID-19 on offers with humans and with computers. Simple regression models predicting offers based on the impact of COVID-19 were statistically significant (Figure 1B: human receivers, *F*(1, 184) = 93.15, p < 0.001, *R*^2^ = 0.336, *B*_0_ = 43.52, *B*_*Covid-19*_ = 15.20; Figure 1C: computer receivers, *F*(1, 184) = 133.76, p < 0.001, *R*^2^ = 0.421, *B*_0_ = 37.96, *B*_*Covid-19*_ = 19.20). Hence, the results suggest that those impacted by COVID-19 were behaving more altruistically than others and, in particular, with computers.

We then focused on comparing offers with humans vs. computers. We ran a mixed model analysis with COVID-19, counterpart type, and the interaction as predictors and the offer percentage as the target variable. The predictors were set as fixed factors; we used an unstructured repeated covariance type for the residuals, and we used the restricted maximum likelihood estimation method. This analysis confirmed the main effect of COVID-19 (p = 0.001) and revealed a main effect of counterpart type (p < 0.001) and a statistically significant COVID-19 × counterpart interaction (p = 0.025). The interaction indicates that participants were making higher offers to humans than computers, except when COVID-19 impact was high. Overall, thus, the results support our hypothesis that those impacted by COVID-19 were making less of a distinction in their offers between computers and humans.

To gather further insight and facilitate interpretation of the results, we also discretized the continuous COVID-19 scale into three categories: low (below 25^th^ percentile), medium, and high (above 75^th^ percentile). We found that the demographics and geographical distributions for participants in the high COVID-19 category were in line with distributions for the impact of COVID-19 in the US, as measured by the number of confirmed deaths on the day the experiment was run (see Figure S3), which gives us confidence that this construct is indexing COVID-19 impact. The offers for each of these categories are shown in Figure 1E. As can be seen, the offers were higher with higher COVID-19 impact, reinforcing the finding that those impacted by COVID-19 were being more altruistic. We then created a new dependent variable measuring the difference in return to humans and computers—which we call the bias in favor of humans—as shown in Figure 1F. We ran an ANOVA on this measure to understand the relative impact of COVID-19 on offers with computers vs. humans. The analysis revealed a main effect of categorical COVID-19 (*F*(2, 183) = 7.10, p = 0.001, partial *η*^*2*^ = 0.072, Figure 1D). Post-hoc tests with a Bonferroni correction revealed that the bias in favor of humans for participants in the high COVID-19 category was lower than that for participants in the low COVID-19 category (p = 0.001).

**Figure 2:**
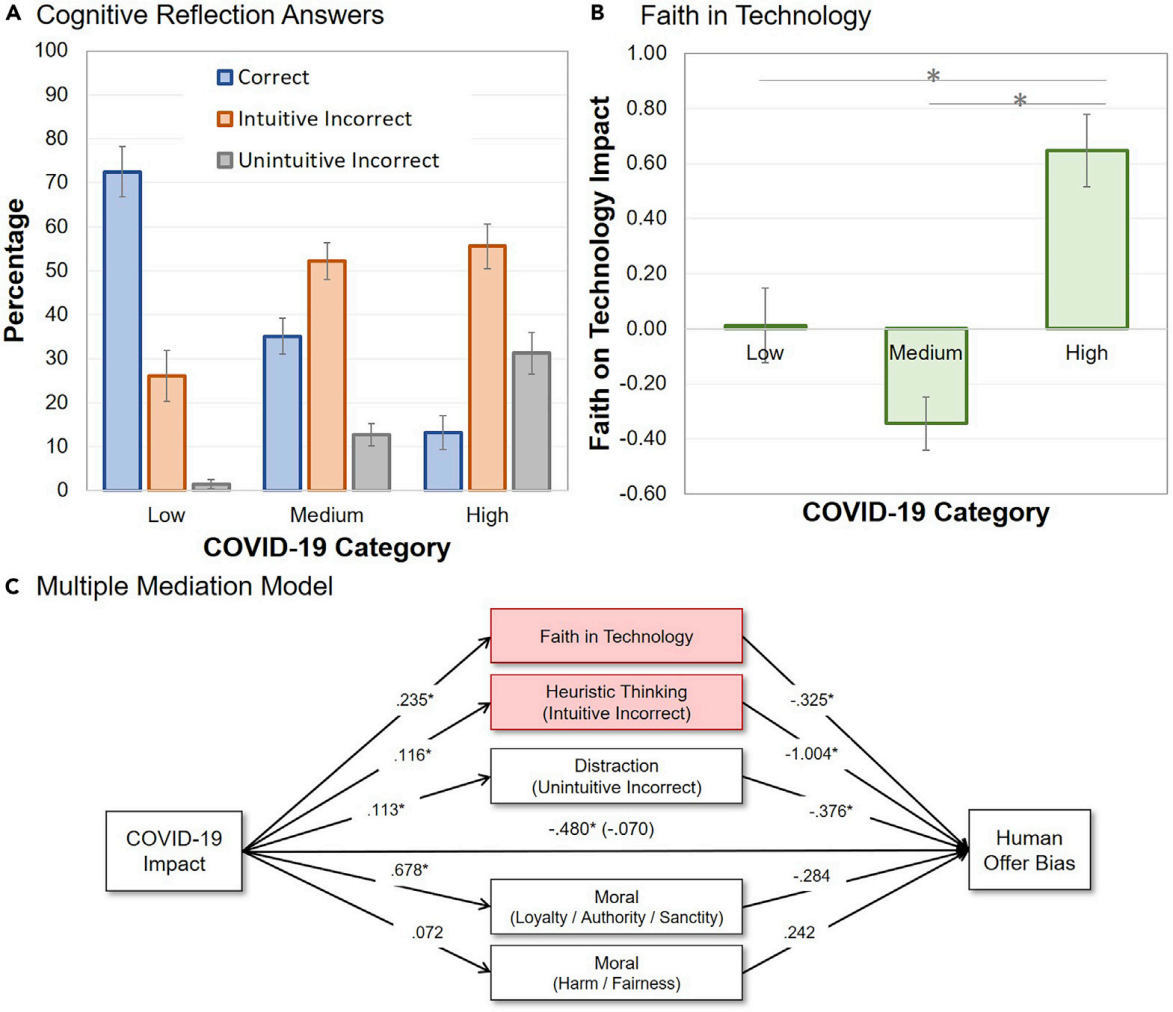
Experimental results for the dictator game (A) Distribution of answers to the cognitive reflection scale. Error bars correspond to standard errors. (B) Participants in the high COVID-19 category showed higher faith in technology than others. Error bars correspond to standard errors. (C) The multiple mediation analysis. Left arrows connecting the independent variable (IV) to mediators indicate the direct effect of the IV on the mediators (typically called *a* path). Right arrows connecting the mediators to the dependent variable (DV) indicate the direct effect of the mediator on the DV (typically called *b* path). The arrow connecting the IV to the DV indicates the total effect of the IV on the DV (typically called *c* path) and, in parenthesis, the direct effect of the IV on the DV (typically called *c’*). Multiple mediation occurs when the total effect is significant, but the direct effect is not, suggesting that (some of) the mediators account for the effect (Preacher and Hayes, 2008). ∗p < 0.05.

Why were participants impacted by COVID-19 being altruistic to computers? To get insight on the mechanism causing the effect, we ran a multiple mediation analysis (Preacher and Hayes, 2008) and considered several possible mediators. A multiple mediation analysis is a statistical technique that helps establish causality by determining if certain mediators (e.g., heuristic thinking) account for the effect of an independent variable (e.g., COVID-19) on a dependent variable (e.g., bias). First, we looked at “heuristic thinking” as measured by incorrect intuitive answers in the cognitive reflection scale. Figure 2A shows the distributions for incorrect answers but also correct and unintuitive incorrect answers for each COVID-19 category. We ran ANOVAs which showed main effects of COVID-19 category on all measures (correct answers: *F*(2, 183) = 32.52, p < 0.001, partial *η*^*2*^ = 0.262; intuitive incorrect: *F*(2, 183) = 33.51, p < 0.001, partial *η*^*2*^ = 0.551; and unintuitive incorrect: *F*(2, 183) = 18.84, p < 0.001, partial *η*^*2*^ = 0.171)—indicating that participants in the high COVID-19 category made more unintuitive incorrect answers than participants in the low COVID-19 category. However, the analysis also revealed that participants in the high COVID-19 category made more unintuitive incorrect answers than participants in the low COVID-19 category, which may indicate that they were distracted. This motivated us to include a second possible mediator—which we called “distraction”—based on the number of unintuitive incorrect answers.

Our third mediator was the “faith in technology” scale. We subjected this scale to a principal component analysis to reduce it to a single factor (Cronbach α = 0.746; see details in Table S2). Figure 2B shows this distribution for each COVID-19 category. An ANOVA showed a main effect (*F*(2, 183) = 18.38, p < 0.001, partial *η*^*2*^ = 0.167) with participants in the high COVID-19 category showing higher faith in technology than participants in the low and medium COVID-19 category. Finally, our fourth mediator was the Moral Foundations Scale. We also subjected this scale to a principal component analysis which resulted in two factors (Graham et al., 2013): loyalty/authority/sanctity (Cronbach α = 0.889) and harm/fairness (Cronbach α = 0.741; see Table S3 for details). ANOVAs showed main effects (loyalty/authority/sanctity: *F*(2, 183) = 61.04, p < 0.001, partial *η*^*2*^ = 0.400; harm/fairness: *F*(2, 183) = 11.63, p < 0.001, partial *η*^*2*^ = 0.113), with participants in the high COVID-19 category showing higher morality scores than participants in the low and medium COVID-19 category. Table S4 shows correlations between the COVID-19 scale and the other scales.

The mediation analysis revealed that the effect of COVID-19 impact was fully mediated (i.e., caused) by increased heuristic thinking (indirect effect: −0.116, p = 0.016) and increased faith in technology (indirect effect: −0.077, p = 0.041), with the total effect (−0.480, p < 0.001) becoming statistically non-significant once the effect of the mediators was accounted for (direct effect: −0.070, p = 0.680) (see Table S5 for bootstrapping confidence intervals). In particular, the indirect effects of distraction (−0.043, p = 0.485) and moral foundations (loyalty/authority/sanctity: −0.193, p = 0.077; harm/fairness: 0.017, p = 0.378) were not statistically significant.

## Discussion

As autonomous technology—e.g., robots, self-driving cars, virtual personal assistants—becomes increasingly available, its adoption and success hinges on the ability to promote collaboration with humans. In this paper, we present insight on mechanisms shaping how people make decisions with machines, with subsequent practical consequence for the design of such technology. This insight was enabled by an unprecedented event—the COVID-19 global pandemic—which is impacting the way people make decisions, at least in the short term, but possibly in the longer term. Our results indicate that, in a dictator game experiment, participants that were impacted by COVID-19 (as measured by a PTSD scale) were being considerably more altruistic with machines than those that were not. The effect, as suggested by a mediation analysis, was explained by increased heuristic thinking, likely motivated by cognitive demands due to the pandemic, and perceptions of increased importance for the role of technology to the future, likely motivated by changes in lifestyle caused by the pandemic.

Increased altruism during the COVID-19 pandemic is broadly in line with prior findings associating natural disasters with increased reciprocity, trust, and altruism (Calo-Blanco et al., 2020; Cassar et al., 2017; Toya and Skidmore, 2014; Whitt and Wilson, 2007). Our findings suggest that the more individuals were impacted by COVID-19, the more likely they were to behave altruistically with others, including non-human others (see the supplemental information for a pilot study suggesting that this effect may also extend to reciprocity). Negative impact on social behavior, typically due to competition for scarce resources following disasters (Carlin et al., 2014; Hsiang et al., 2013), may have been avoided in this case due to the government's quick response in terms of financial aid to the population and businesses—similarly to the impact government programs have had in the past (Bruneau et al., 2003).

We present insight on the cause for this effect, with clear theoretical implications for our understanding of human behavior with machines. Our mediation analysis indicates that heuristic thinking and faith in technology fully mediated the effect of COVID-19 impact on decisions with machines. This reinforces but more importantly extends the Computers as Social Actors theory of Reeves and Nass (1996). This theory, which is very influential in human computer and robot interaction research, has argued that people heuristically treat machines like people; however, this body of research has not drawn explicit links to cognitive science research on reflective versus heuristic thinking (e.g., Frederick, 2005). Besides making these links, our findings exclude distraction as a possible mediator for this effect, emphasizing that heuristic thinking, not the absence of thinking, explains prosocial behavior toward machines. Moreover, prior research reveals important differences in the decisions people make with machines, when compared to humans (de Melo and Terada, 2019; Gallagher et al., 2002; McCabe et al., 2001; Rilling et al., 2002; Sanfey et al., 2003). Our results show that heuristic thinking can help mitigate these differences, closing the gap on distinctions people make between humans and machines. More broadly, these findings seem in line with predictions from the social heuristics theory, whereby encouraging intuitive thinking, in contrast to deliberation, can lead to increased cooperation in non-strategic settings (Rand, 2016; Rand et al., 2014). Our work extends this prior work by showing that this effect extends to interaction with machine counterparts.

The second mediator—faith in technology—suggests possible longer effects of COVID-19 on human behavior with machines. This scale measured participants' expectations about the role technology will play in improving quality of life in the future. The results indicated that those impacted by COVID-19 rated higher on this scale, which may reflect positive experiences with technology as they were forced to engage remotely in their social and professional life. Moreover, this scale mediated the effect of COVID-19 on behavior with machines, suggesting that improved perceptions about the value of technology can lead to more favorable decisions with machines.

The results presented here have practical implications for the design of technology and autonomous machines. Prior research indicates that people often consider the others' welfare when making decisions (Axelrod, 1984; Kollock, 1998; Rand and Nowak, 2013). Here, we show that it is possible to motivate this type of social consideration when engaging with machines by encouraging users to think heuristically and draw on their human-human interaction experiences when engaging in human-machine interaction (Reeves and Nass, 1996). However, whereas the present effect was caused by stress due to the COVID-19 pandemic, it is possible to encourage heuristic thinking in healthier ways, such as by expressing emotion (de Melo and Terada, 2020; de Melo et al., 2014) or through cultural cues in machines (de Melo and Terada, 2019). As the results further show, this approach can mitigate fundamental biases users show toward machines (de Melo and Terada, 2019; Gallagher et al., 2002; McCabe et al., 2001; Rilling et al., 2002; Sanfey et al., 2003) which, if left unaddressed, constitute important barriers to the adoption of technology. On the cautionary side, it may not always be valuable for users to treat machines as if they were social actors—e.g., to manage expectations about the machine's capabilities or to avoid exploitation. In the present case, the results suggest that those impacted by the COVID-19 pandemic may be particularly susceptible to be socially influenced by machines. Given the disproportional impact of COVID-19 on economically vulnerable groups, this highlights the need for ethical guidelines and regulations to ensure the altruism shown to machines is well deserved. Generally, the same theory would suggest that, when it is important to control the social expectations about machines, we can discourage heuristic thinking by motivating users to think more deliberatively through the interaction. Overall, the judicious application of the theory discussed here can lead to the development of technology that is able to build collaboration with humans and, ultimately, be successfully adopted in practice.

### Limitations of the study

The present study has limitations that introduce opportunities for future work. Even though we considered several possible mediators for the effect of COVID-19 on behavior with machines, it is possible that there are other relevant factors at play. For instance, individual stress propensity, level of education, and socio-economic status could simultaneously make individuals susceptible to engage in heuristic thinking and being impacted by COVID-19. Future work should, thus, study these factors to help understand their relative importance to the effect. The present study focused on altruism, but there are other relevant forms of social consideration—such as reciprocity, trust, and fairness—that may shape collaboration between humans and machines. Follow-up work should complement the work presented here with a study of the relationship between heuristic thinking and these constructs and corresponding impact on human behavior. The sample of participants for this study was collected during the initial stage of the pandemic in the US; however, it would be worth comparing these results with samples taken at different stages, which may be subject to additional sources of variation (e.g., pandemic fatigue). It should also be worth comparing this study to data collected in other geographical regions in the world. Finally, whereas the present study reports phenomena that occurred in the context of the COVID-19 global pandemic, it is relevant to confirm and understand if there are differences in the way people think and behave under more normal circumstances; in particular, other manipulations for heuristic thinking should be explored.

### Resource availability

#### Lead contact

Further information and requests for resources should be directed to and will be fulfilled by the lead contact, Celso M. de Melo (celso.miguel.de.melo@gmail.com).

#### Materials availability

This study did not generate new unique reagents.

#### Data and code availability

The article includes with the supplemental materials all experimental data collected and analyzed during the studies discussed in the paper. The code supporting the current study has not been deposited in a public repository because it includes proprietary and licensed software but some materials are available from the corresponding author on request.

## Methods

All methods can be found in the accompanying transparent methods supplemental file.

## Supplemental information

Document S1. Transparent methods, Figures S1–S3, and Tables S1–S5

Data S1. Experimental data for the dictator experiment, related to Figure 1

Data S2. Experimental data for the pilot experiment, related to Figure 1

Data S3. Screenshots for experiment survey, related to Figure 1

## Footnotes

Supplemental information can be found online at https://doi.org/10.1016/j.isci.2021.102228.
