# Supplementary material for: Heuristic thinking and altruism toward machines in people impacted by COVID-19
Source: iScience. 2021 Feb 23;24(3):102228. doi: 10.1016/j.isci.2021.102228 (PMC7901281; doi:10.1016/j.isci.2021.102228)
Supplement: Document S1. Transparent methods, Figures S1–S3, and Tables S1–S5 [file mmc1.pdf]

iScience, Volume *24*

Supplemental information

Heuristic thinking and altruism toward machines in people impacted by COVID-19

Celso M. de Melo, Jonathan Gratch, and Frank Krueger

Transparent Methods

Participant sample. Participants for the experiment were recruited from Amazon Mechanical Turk. All participants were recruited from the United States and had an approval rate, based on prior work in this pool, of at least 95%. To estimate the sample size for the experiment, we followed the power calculations proposed by Jacob Cohen and implemented in G*Power[1] —a software that is often used by behavioral researchers. We estimated sample size for a 3 × 2 mixed factorial design: categorical Covid (low vs. medium vs. high) × counterpart (human vs. computer). For a small effect size (Cohen's $f = 0.20$), $\alpha = .05$, and statistical power of 0.95, the recommended total sample size was 177 participants, which rounds up to 180 participants to keep the distribution even across cells. When recruiting from this pool, it is common for some participants to fail to successfully complete the task or otherwise make data entry errors. To account for that, we increased the target sample size experiment to 190 participants. The sample was collected on May 6, 2020. As noted in the main text, this was while most of the United States was still under strict lockdown measures. Average completion time for the experiments was about 45 minutes.

In practice, we recruited 186 participants from 38 states with the following demographics distribution: gender (73.1% males; 26.3% females; 0.6% did not disclose); age distribution (18 to 21 years, 0.5%; 22 to 34 years, 54.8%; 35 to 44 years, 25.8%; 45 to 54 years, 10.2%; 55 to 64 years, 8.1%; over 64 years, 0.6%); and ethnicity distribution (Caucasian, 54.8%; African American, 25.8%; East Indian, 1.6%; Hispanic or Latino, 12.4%; Southeast Asian, 5.4%).

[1] https://www.psychologie.hhu.de/arbeitsgruppen/allgemeine-psychologie-und-arbeitspsychologie/gpower.html (Last accessed: May-31, 2020)

Covid-19 impact demographics. To measure the Covid-19 impact, we used the abbreviated Checklist-Civilian 6-item scale for measuring posttraumatic stress disorder (PTSD)[2]. It asks how troubled the respondent has been by a serious of symptoms associated with PTSD (e.g., have you been bothered by repeated, disturbing memories, thoughts, or images?). Following standard recommendations, we adapted the instructions to refer to a specific event (the Covid-19 pandemic) and time frame (bothered by these symptoms in the last month). For the formal analyses presented in the main text, we ran a principal component analysis, with varimax rotation, to reduce the scale to a single factor that better explains the variance in the data. As shown in Table S1, the loadings were similar across experiments. The distribution for the Covid-19 factor is shown in Figure S1. For some follow-up analyses, we further categorized the Covid-19 factor scale into three categories: low (below 25th percentile), medium, and high (above the 75th percentile). The state distribution for high Covid-19 participants was in line with the Covid-19 death count in the United States (Fig. S3) —for instance, many of the high Covid-19 participants came from California, New York, and Florida, which were also some of the states with the highest death counts.

Covid-19 impact and absence of political bias. We failed to find evidence that political bias influenced the results. To get insight into possible political bias in participants' responses to the Covid-19 scale, we created two variables: (a) state Republican advantage, which represents the Republican advantage, in terms of percentage of the total vote, in the 2016 United States election (e.g., for Florida this was 1.3%); and (b) state color (blue vs. red), based on the results of the 2016 election. We found no correlation between Covid-19 and Republican advantage (r^2 = -0.006). We then looked at regression models for the main

---

[2] Lang, A. and Stein, M. An abbreviated PTSD checklist for use as a screening instrument in primary care. Behav. Res. Ther. *43*, 585-594 (2005).

dependent variables using as predictors Covid-19 and Republican advantage. We found that Republican advantage was not a statistically significant predictor for any of the variables — for instance, for offers with humans in the dictator game, $B_{Republican} = -0.002$, $p = 0.847$. We, then, ran categorical Covid-19 × state color ANOVAs on the main dependent variables and found no main effects or interactions of state color.

We also asked self-reported political orientation using an existing scale[3]. This consisted of three questions on a 7-point Likert scale (1, *Very conservative*, to 7, *Very liberal*): How would you describe your political orientation on (a) economic issues, (b) social issues, and (c) overall. We ran a principal component analysis on this measure, which resulted in the expected single factor. This scale was correlated with Covid-19 ($r^2 = 0.319$, $p < 0.001$). However, when including this predictor in the regression models for offers with humans, offers with computers, and offer bias, Covid-19 remained a statistically significant predictor: offers with humans, $B_{Covid\text{-}19} = 1.676$ ($p < 0.001$), $B_{Liberal} = 0.487$ ($p = 0.020$); offers with computers, $B_{Covid\text{-}19} = 2.148$ ($p < 0.001$), $B_{Liberal} = 0.464$ ($p = 0.020$); offer bias, $B_{Covid\text{-}19} = -0.472$ ($p < 0.001$), $B_{Liberal} = -0.024$ ($p = 0.853$).

Full anonymity. The experiment was fully anonymous for participants. This was accomplished by giving counterparts anonymous names and avoiding collection of information that can identify participants. To maintain anonymity with experimenters, we relied on the anonymity system provided by Mechanical Turk. In this system, researchers cannot identify participants, unless they ask for identifiable information, which we did not. This procedure minimizes reputation effects, such as concerns for retaliation for decisions made in the experiment.

---

[3] B. Lassetter, R. Neel. Malleable liberals and fixed conservatives? Political orientation shapes perceived ability to change. *J. Exp. Soc. Psychol. 82*, 141-151 (2019).

Financial incentives. Participants were paid $2.00 for taking part in the experiment, which is a typical amount for this online pool. Moreover, they had the opportunity to earn more money according to their performance in the task. Each point earned in the task was converted to a ticket for a lottery worth $30.00. Participants were instructed and quizzed for comprehension on game instructions.

Pilot Experiment

We first identified the effect of Covid-19 impact on decisions with machines in an experiment where participants engaged in a trust game[4] with humans and computers. The trust game is a two-player sequential reciprocal game, where a sender (or trustor) is given an initial endowment of money —in our case, 12 tickets for a lottery worth $30. The sender decides how many tickets to send to the receiver (or trustee), and this investment is multiplied by 3. The receiver then decides how many tickets to return to the sender. Rational theory argues the receiver has no incentive to return any ticket and, thus, the sender should also have no incentive to send any ticket. If the sender sends any amount greater than zero, then the sender is argued to have shown trust towards the receiver. The receiver is said to reciprocate if the returned amount is greater than the amount sent.

In this experiment, participants engaged in 12 trials of the trust game in the role of the receiver. Participants were instructed that senders would either be other participants or computers. In reality, to maximize experimental control, participants always engaged with computer scripts that followed a fixed sequence of offers: human counterparts —6, 1, 5, 12, 2, and 11 tickets; computer counterparts —6, 2, 5, 11, 1 and 12 tickets.

We recruited 184 participants from Mechanical Turk and used the same measure for Covid-19 as reported in the main text. Simple regression models predicting returns based on

---

[4] Berg, J., Dickhaut, J. and McCabe, K. Trust, reciprocity, and social history. Games Econ. Behav. *10*, 122-142 (1995).

impact of Covid-19 were statistically significant (human senders, $F(1, 182) = 43.92$, $p < 0.001$, $R^2 = 0.194$, $B_0 = 38.95$, $B_{Covid\text{-}19} = 9.53$; computer senders, $F(1, 182) = 45.20$, $p < 0.001$, $R^2 = 0.199$, $B_0 = 34.00$, $B_{Covid\text{-}19} = 11.37$). The results indicated that the higher the impact of Covid-19, the higher the return in the trust game, suggesting that those impacted by Covid-19 reciprocated more. Moreover, given that the slope of the linear fit was higher with computers, the results suggest that those impacted by Covid-19 returned more with computers. To gather further insight, we discretized the continuous Covid-19 scale into three categories: low (below 25th percentile), medium, and high (above 75th percentile). We created a new dependent variable measuring the difference in return to humans and computers —the bias in favor of humans. An analysis of variance (ANOVA) on this variable confirmed a main effect of categorical Covid-19 ($F(2, 181) = 3.87$, $p = 0.023$, partial $\eta^2 = 0.041$). Post-hoc tests with a Bonferroni correction revealed that, for high-Covid participants, the bias in favor of humans tended to be lower than medium-Covid participants ($p = 0.052$) and was lower than low-Covid participants ($p = 0.036$). This experiment, thus, identified an unexpected effect of impact of Covid-19 on behavior with machines. The main text reports a more focused and carefully designed experiment that studies this effect and further provides insight on the mechanism underlying it.

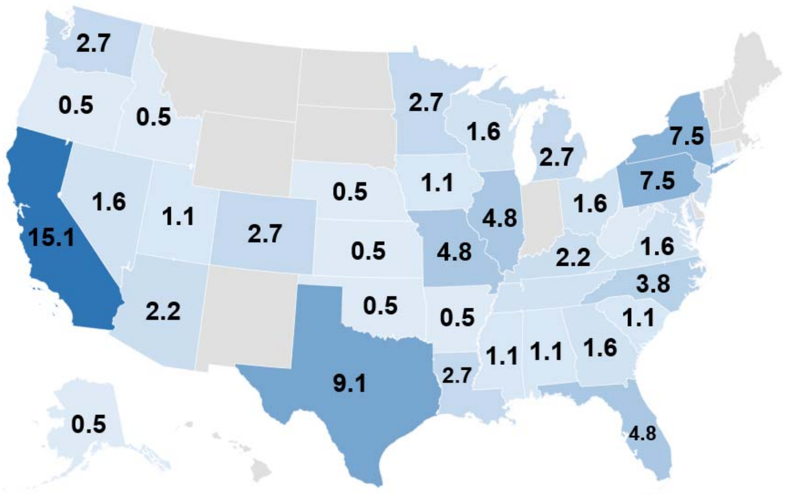

Fig. S1. Participant sample distribution per state (percentage), Related to Figure 1.

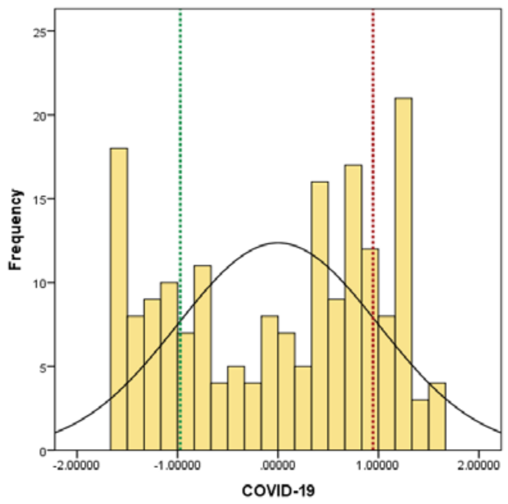

Fig. S2. Covid-19 scale distribution in the participant sample, Related to Figure 1. The green line marks the 25th percentile and the red line the 75th percentile. Participants below the 25th percentile were categorized as “low Covid-19” and above the 75th percentile as “high Covid-19”.

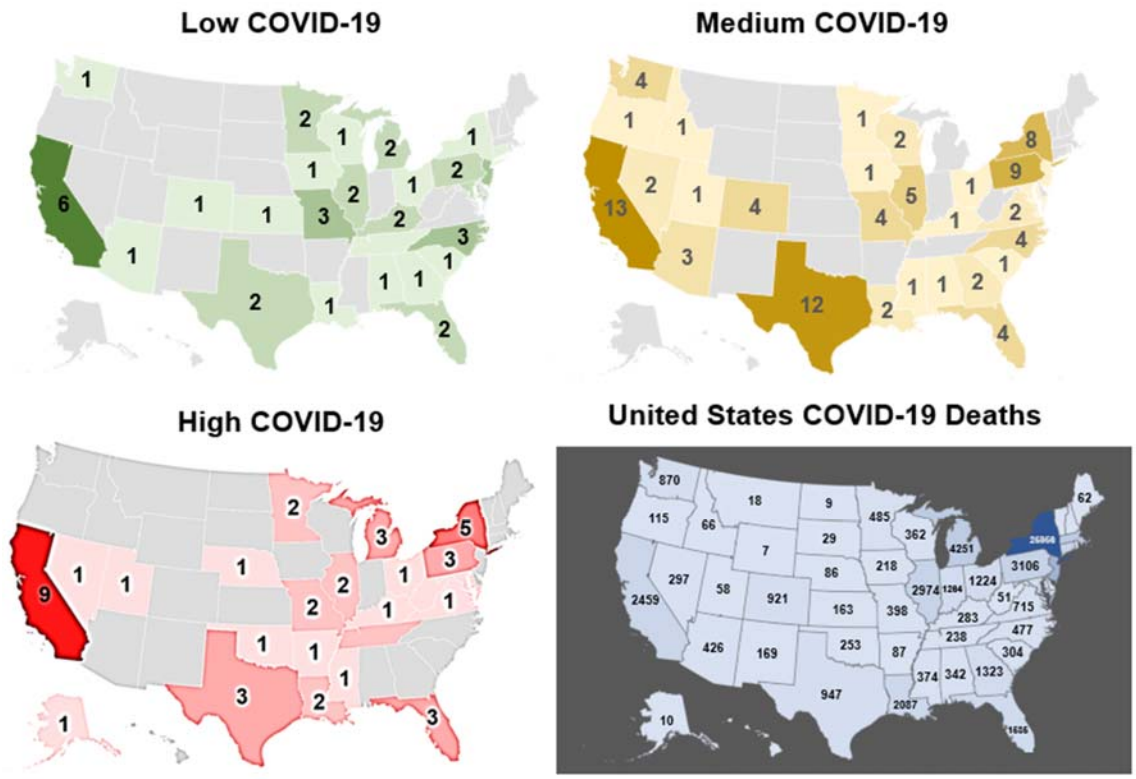

Fig. S3. State distribution for participant sample's Covid-19 impact categories in comparison to the number of deaths in the United States on the day the sample was collected (May-6, 2020), Related to Figure 1.

Table S1. Principal component analysis loading factors for the Covid-19 Scale, Related to Figure 1. The instructions for this scale were: "The Covid-19 pandemic is causing disruption to many people's lives. Below is a list of problems and complaints that people may have in response to the outbreak. Please read each one carefully and indicate how much you have been bothered by that problem in the *last month.*"

Question	Factor Loading
1. Repeated, disturbing memories, thoughts, or images?	.918
2. Feeling very upset when something reminded you of the situation?	.890
3. Avoiding activities or situations that remind you of the situation?	.876
4. Feeling distant or cut off from other people?	.776
5. Feeling irritable or having angry outbursts?	.882
6. Having difficulty concentrating?	.905

Table S2. Principal component analysis loading factors for the Faith in Technology Scale, Related to Figure 2. The instructions for this scale were: "Please indicate how much you agree with the following statements".

Question	Factor Loading
1. Computer technology will change life for the better	0.797
2. I believe computer technology can be developed to help alleviate society's problems	0.714
3. Computer technology can help us understand and control physical, biological and social processes for the benefit of present and future generations	0.695
4. Computer technology advances will solve America's social and economic problems within the next ten years	0.544
5. Computer technology is improving the services available to society	0.807

Table S3. Principal component analysis loading factors for the Moral Foundations Scale, Related to Figure 2. The instructions for this scale were: "Please indicate how much you agree with the following statements".

Question	Factor 1 Loading	Factor 2 Loading
1.Whether or not someone suffered emotionally	0.318	0.723
2.Whether or not some people were treated differently than others	0.125	0.743
3. Whether or not someone's action showed love for his or her country	0.866	-0.004
4. Whether or not someone showed a lack of respect for authority	0.822	0.152
5. Whether or not someone violated standards of purity and decency	0.785	0.120
6. Whether or not someone cared for someone weak or vulnerable	0.239	0.716
7. Whether or not someone did something to betray his or her group	0.794	0.246
8. Whether or not someone did something disgusting	0.805	0.115
9. When the government makes laws, the number one principle should be ensuring that everyone is treated fairly.	-0.091	0.752
10. Respect for authority is something all children need to learn.	0.648	0.182

Table S4. Correlations between the Covid-19 scale and the Cognitive Reflection, Faith in Technology, and Moral Foundations factors, Related to Figure 2. * $p < .05$

	Cognitive Reflection (Correct)	Cognitive Reflection (Intuitive Incorrect)	Cognitive Reflection (Unintuitive Incorrect)	Faith in Technology	Moral (Loyalty / Authority / Sanctity)	Moral (Harm / Fairness)
Covid-19	-.547*	.288*	.433*	.235*	.678*	0.072
Cognitive Reflection (Correct)		-.797*	-.375*	-.195*	-.504*	0.086
Cognitive Reflection (Intuitive Incorrect)			-.261*	0.094	.317*	-0.108
Cognitive Reflection (Unintuitive Incorrect)				.169*	.319*	0.029
Faith in Technology					.324*	.302*
Moral (Loyalty / Authority / Sanctity)						0.000

Table S5. Bootstrapping Analysis of the Statistical Significance of the Indirect Effects for the Multiple Mediation Analysis, Related to Figure 2.

Indirect Effect	Point Estimate	Standard Error	95% Confidence Interval	
			Lower Bound	Upper Bound
Total	-0.410	0.157	-0.748	-0.145
Heuristic Thinking (Intuitive Incorrect)	-0.116	0.045	-0.226	-0.045
Distraction (Unintuitive Incorrect)	-0.043	0.055	-0.163	0.059
Faith in technology	-0.077	0.040	-0.192	-0.022
Moral (Loyalty / Authority / Sanctity)	-0.193	0.134	-0.486	0.033
Moral (Harm / Fairness)	0.017	0.022	-0.009	0.087
