# Supplementary material for: Heuristic thinking and altruism toward machines in people impacted by COVID-19
Source: iScience. 2021 Feb 23;24(3):102228. doi: 10.1016/j.isci.2021.102228 (PMC7901281; doi:10.1016/j.isci.2021.102228)
Supplement: Data S3. Screenshots for experiment survey, related to Figure 1 [file mmc4.zip › Data S3.pdf]

Introduction

Welcome to Our Study!

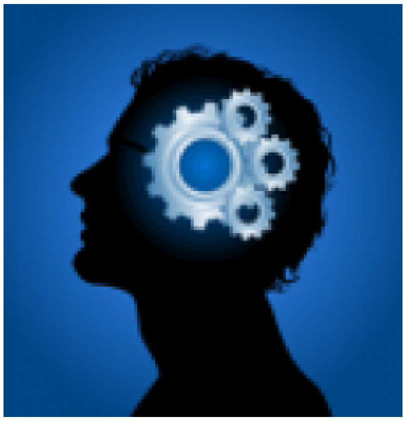

Today, you will engage in one decision making task. The goal of this experiment is to study how people make decisions online. This survey is anonymous and your time commitment is expected to be no more than 45 minutes.

Please proceed now to the next screen to begin the survey.

>>

Exclude Repeat Participants

Minimum Requirements

Please confirm that you qualify for this study.
Here's your Worker ID: **participantX**.

[Click this link to open the task in a separate tab.]

When you finish, you will receive a **CODE**. Please return to this tab and insert it below to proceed:

>>

You are good to go!

Please go back to the survey and insert this CODE to proceed:

6951_PR

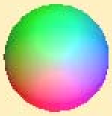

This software checks if this participant has run one of our related studies in the recent past. Participants that ran related studies were excluded.

Pre-Questionnaire: Social Value Orientation

Pre-Questionnaire 1/2

Imagine you will be making a series of decisions about allocating resources between you and another person. For each of the following questions, please indicate the distribution you prefer most by marking the respective position along the midline. You can only make one mark for each question.

Imagine the numbers represent money. In the example below, a person has chosen to distribute money so that he/she receives 50 dollars, while the anonymous other person receives 40 dollars.

There are no right or wrong answers, this is all about personal preferences.

Example:

You receive	30	35	40	45	50	55	60	65	70
Other receives	80	70	60	50	40	30	20	10	0

You 50

Other 40

	Option 1	Option 2	Option 3	Option 4	Option 5	Option 6	Option 7	Option 8	Option 9
You receive	85	85	85	85	85	85	85	85	85
Other receives	85	76	68	59	50	41	33	24	15

1 2 3 4 5 6 7 8 9

A)

	Option 1	Option 2	Option 3	Option 4	Option 5	Option 6	Option 7	Option 8	Option 9
You receive	85	87	89	91	93	94	96	98	100
Other receives	15	19	24	28	33	37	41	46	50

1 2 3 4 5 6 7 8 9

B)

You receive

50 54 59 63 68 72 76 81 85

Pre-Questionnaire: Demographics

Pre-Questionnaire 2/2

Please answer the following questions about yourself.

1. Age?

[▾]

2. Gender?

- ○ Male
- ○ Female
- ○ Other

3. Country of Citizenship?

[▾]

4. Which of the following best matches your ethnicity?

- ○ African American
- ○ Caucasian
- ○ East Indian (e.g., India, Pakistan)
- ○ Hispanic or Latino
- ○ Southeast Asian (e.g., China)

5. Profession?

[ ]

>>

Dictator Game Instructions 1/3

TASK INSTRUCTIONS

You are going to engage in a 2-player decision task. There are two players: Sender and Receiver. Sender is initially given 12 tokens. Sender is then given the opportunity to send tokens to the Receiver (from 0 to 12 tokens). The amount sent by the Sender is final and once the offer is made the game is over.

1) There are two players: Sender and Receiver.
Sender is given 12 tokens

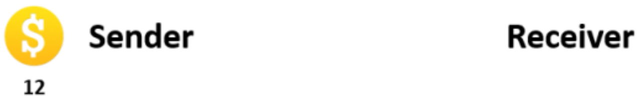

2) Sender is given the opportunity to send *x* tokens to Receiver

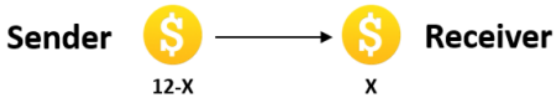

3) The game is over

For instance, imagine the Sender sends 2 tokens. The Receiver will get 2 tokens and the Sender will keep 10 tokens.

LOTTERY
The tokens you earn matter because they will give you a chance to win real money from a research grant. The greater your tokens, the greater your chances of earning a $30 lottery. This lottery will be conducted at the end of the month, after all participant answers have been collected. This is real money and will be paid through Mechanical Turk to the winner.

QUIZ

Before moving forward, please answer a few questions to make sure you understand the instructions. If you have any questions, please refer back to the instructions at the top of the page.

1. If Sender sends 4 tokens, how many will each player get?
   - ◯ Sender: 4 tokens Receiver: 8 tokens
   - ◯ Sender: 4 tokens Receiver: 4 tokens
   - ◯ Sender: 8 tokens Receiver: 4 tokens

2. Why is it important to earn tokens?
   - ◯ Because it increases your chances for a $10 lottery
   - ◯ Because it increases your chances for a $15 lottery
   - ◯ Because it increases your chances for a $30 lottery

Participant must reply correctly to quiz questions in order to proceed.

Dictator Game Instructions 2/3

YOU WILL ENGAGE IN THE TASK 12 TIMES WITH DIFFERENT COUNTERPARTS

You will engage in the task 12 times, each time with a different counterpart. In all games, you will engage as Sender.

Each task will have an *independent* lottery - i.e., there will be 12 lotteries (one per game) and the tokens you earn in one game will not influence the lottery in the other.

QUIZ

Before moving forward, please answer a few questions to make sure you understand the instructions. If you have any questions, please refer back to the instructions at the top of the page.

1. How many times will you engage in the task?

- ◯ Once
- ◯ 12x (as Sender)

2. Are the lotteries for the tasks independent?

- ◯ No
- ◯ Yes, the tokens you earn in one game will not affect the lottery in the other

>>

Dictator Game Instructions 3/3

Your Counterparts Will Be Other MTurkers and Computer Programs

Today, you will engage in decision-making tasks with *other MTurkers* and with *computer programs*. All participants will be anonymous, i.e., you won't be able to identify your counterparts and your counterparts will not be able to identify you. For other MTurkers, we will refer to them as "anonymous" and to computer programs as "computers". Computer programs will follow deterministic scripts to make their decisions.

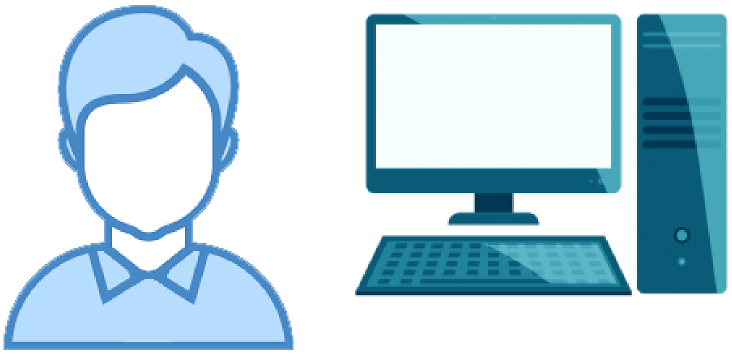

Quiz

Before moving forward, please answer a few questions to make sure you understand the instructions. If you have any questions, please refer back to the instructions at the top of the page.

1. Who will engage with you in the tasks?

- ◯ Other MTurkers and computer programs
- ◯ Computer programs only
- ◯ Other MTurkers only

2. Will you be able to identify your counterparts?

- ◯ No, the task will be anonymous
- ◯ Yes

>>

Dictator Game 1/2

Summary

In sum, in today's tasks:

- You will engage in the task 12x, every time with a different counterpart
- Your counterparts will be other MTurkers and with computer programs

Please proceed now to the next screen to start the task.

>>

Dictator Game 2/2

Task

Here's your Worker ID: **participantX**
You will need it to start the task.

Click this link to open the task in a separate tab.

When you finish, you will receive a **CODE**. Please return to this tab and insert it below to proceed:

>>

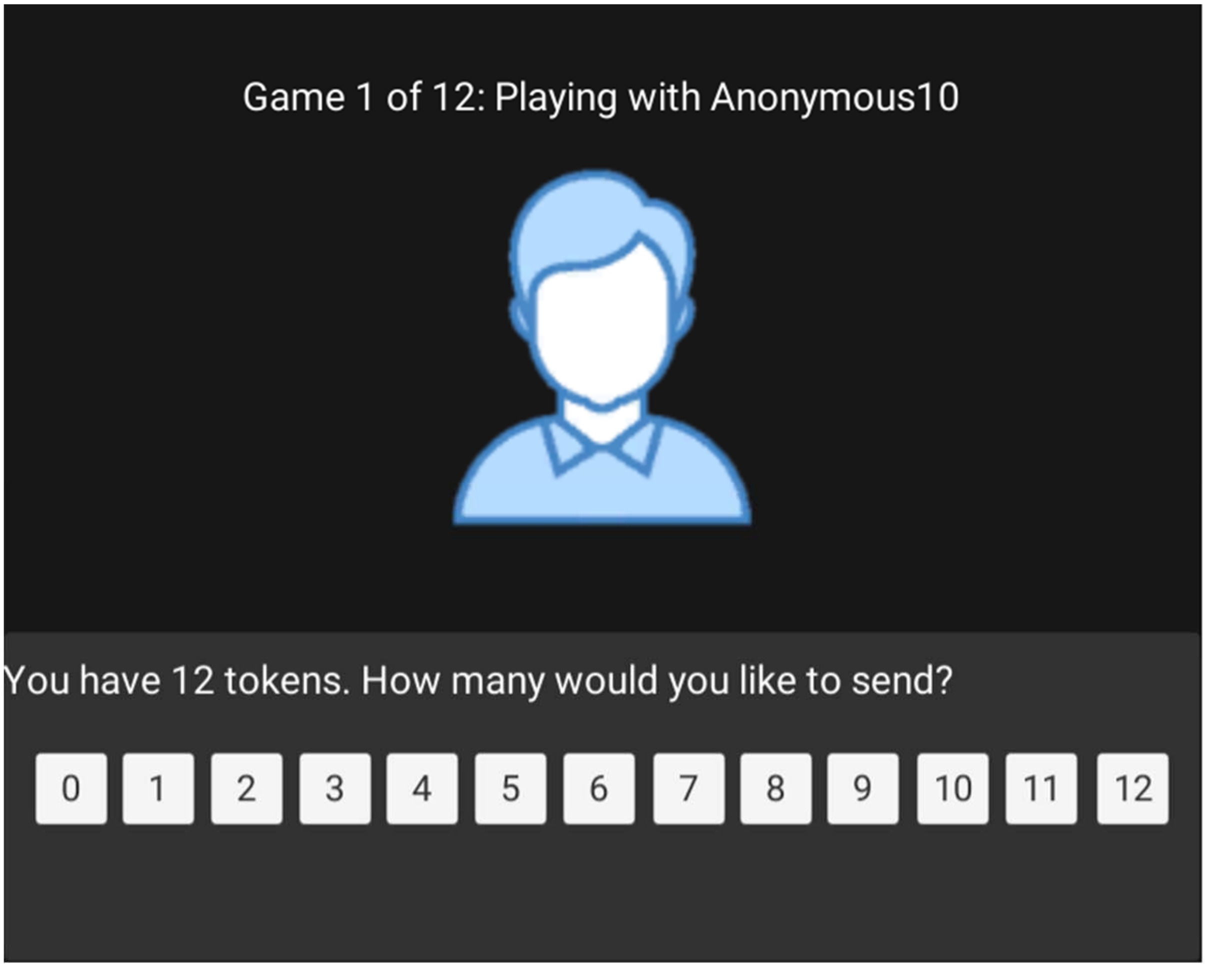

Post-Questionnaire: Covid PTSD Scale

The COVID-19 pandemic is causing disruption to many people's lives. Below is a list of problems and complaints that people may have in response to the outbreak. Please read each one carefully and indicate how much you have been bothered by that problem in the *last month*.

	1 - Not at all	2	3 - moderately	4	5 - extremely
**1.** Repeated, disturbing memories, thoughts, or images?	○	○	○	○	○
**2.** Feeling very upset when something reminded you of the situation?	○	○	○	○	○
**3.** Avoiding activities or situations that remind you of the situation?	○	○	○	○	○
**4.** Feeling distant or cut off from other people?	○	○	○	○	○
**5.** Feeling irritable or having angry outbursts?	○	○	○	○	○
**6.** Having difficulty concentrating?	○	○	○	○	○

>>

Post-Questionnaire: Cognitive Reflection Test

Please answer the following questions.

1. A bat and a ball cost $1.10 in total. The bat costs $1.00 more than the ball. How much does the ball cost (in cents)?

2. If it takes 5 machines 5 minutes to make 5 widgets, how long would it take 100 machines to make 100 widgets (in minutes)?

3. In a lake, there is a patch of lily pads. Every day, the patch doubles in size. If it takes 48 days for the patch to cover the entire lake, how long would it take for the patch to cover half the lake (in days)?

>>

Post-Questionnaire: Faith in Technology

Please indicate how much you agree with the following statements:

	1 - I strongly disagree	2	3	4	5 - I strongly agree
**1.** Computer technology will change life for the better	○	○	○	○	○
**2.** I believe computer technology can be developed to help alleviate society's problems	○	○	○	○	○
**3.** Computer technology can help us understand and control physical, biological and social processes for the benefit of present and future generations	○	○	○	○	○
**4.** Computer technology advances will solve America's social and economic problems within the next ten years	○	○	○	○	○
**5.** Computer technology is improving the services available to society	○	○	○	○	○

>>

Post-Questionnaire: State of Residence

1. In which state do you currently reside?

2. How would you describe where you live in?

1- Very rural	2	3	4	5 - Very urban
○	○	○	○	○

3. In which city do you currently reside? (Optional)

>>

Post-Questionnaire: Moral Foundations Questionnaire

1. When you decide whether something is right or wrong, to what extent are the following considerations relevant to your thinking? Please rate each statement using this scale:

0 - not at all relevant (This consideration has nothing to do with my judgments of right and wrong)
1 - not very relevant
2 - slightly relevant
3 - somewhat relevant
4 - very relevant
5 - extremely relevant (This is one of the most important factors when I judge right and wrong)

	0	1	2	3	4	5
**a.** Whether or not someone suffered emotionally	○	○	○	○	○	○
**b.** Whether or not some people were treated differently than others	○	○	○	○	○	○
**c.** Whether or not someone's action showed love for his or her country	○	○	○	○	○	○
**d.** Whether or not someone showed a lack of respect for authority	○	○	○	○	○	○
**e.** Whether or not someone violated standards of purity and decency	○	○	○	○	○	○
**f.** Whether or not someone cared for someone weak or vulnerable	○	○	○	○	○	○
**g.** Whether or not someone did something to betray his or her group	○	○	○	○	○	○
**h.** Whether or not someone did something disgusting	○	○	○	○	○	○

2. Please read the following sentences and indicate your agreement or disagreement:

0 - strongly disagree
1 - moderately disagree
2 - slightly disagree
3 - sligthly agree
4 - moderately agree
5 - strongly agree

	0	1	2	3	4	5
**a.** When the government makes laws, the number one principle should be ensuring that everyone is treated fairly.	○	○	○	○	○	○
**b.** Respect for authority is something all children need to learn.	○	○	○	○	○	○

>>

Post-Questionnaire: Political Orientation

How would you describe your political orientation on the following issues:

	1 - Very conservative	2	3	4 - Moderate	5	5	7 - Very liberal
**1.** Economic issues	○	○	○	○	○	○	○
**2.** Social issues	○	○	○	○	○	○	○
**3.** Overall	○	○	○	○	○	○	○

>>
